# Datasets on the production and perception of underlying and epenthetic glottal stops in Maltese

**DOI:** 10.1016/j.dib.2020.105543

**Published:** 2020-04-14

**Authors:** Holger Mitterer, Sahyang Kim, Taehong Cho

**Affiliations:** a Department of Cognitive Science, University of Malta, Msida, Malta; b Department of English Education, Hongik University, Seoul, Republic of Korea; c Hanyang Institute for Phonetics and Cognitive Science, Department of English Language and Literature, Hanyang University, Seoul, Republic of Korea

**Keywords:** Maltese glottal stops, Phonemic versus epenthetic, Two-alternative forced choice, Eye-tracking data, Gating data, Segmental versus suprasegmental processing, Spoken word recognition, Prosody

## Abstract

This article provides some supplementary analysis data of speech production and perception of glottal stops in the Semitic language Maltese. In Maltese, a glottal stop can occur as a phoneme, but also as a phonetic marker of vowel-initial words (as in the case with Germanic languages like English). Data from four experiments are provided, which will allow other researchers to reproduce the results and apply their own data-analysis techniques to these data for further data exploration. A production experiment (Experiment 1) investigates how often the glottal marking of vowel-initial words occurs (causing vowel-initial words to be ambiguous with words starting with a glottal stop as a phoneme) and whether the glottal gesture for this marking can be differentiated from an underlying (phonemic) glottal stop in its acoustic properties. Experiments 2 to 4 investigate how and to what extent Maltese listeners perceive glottal markings as lexical (phonemic) or epenthetic (phonetic), using a two-alternative forced choice task (Experiment 2), a visual-world eye tracking task with printed target words (Experiment 3) and a gating task (Experiment 4). A full account of theoretical consequences of these data can be found in the full length article entitled “The glottal stop between segmental and suprasegmental processing: The case of Maltese” [1].

Specifications table

**Table:** 

Subject	Psychology: Experimental and Cognitive Psychology
Specific subject area	Psycholinguistics; Speech Perception; Spoken Word Recognition; Speech Prosody
Type of data	The raw data as UTF-8 encoded text for statistical analyses; Speech files in wave format (wav) for auditory stimuli
How data were acquired	The acoustic speech data (Experiment 1) were obtained with SpeechRecorder [2] and a Focusrite CM25 condenser microphone connected to a Focusrite 2i2 USB audio interface installed on a standard PC. The data of the two-alternative forced choice (2AFC) (Experiment 2) were obtained using PsychoPy [3] (version 1.84), with sounds presented through Logitech Z 150 speakers. The eye-tracking and the gating data (Experiments 3 and 4) were obtained using Experiment Builder with sounds presented through headphones. Eye movements were tracked with an Eyelink 1000 eye-tracker in a desktop mode at a frequency of 500 Hz.
Data format	Raw and processed
Parameters for data collection	Production Data (Experiment 1): The target word is vowel-initial versus glottal stop initial; and the preceding word ends on a vowel or a consonant (=hiatus or not).
	The 2AFC data (Experiment 2): The target word differs in the strength of glottal gesture; and the preceding word is lengthened or not (the presence/absence of final lengthening)
	The eye-tracking and the gating data (Experiments 3 and 4): The target word starts with a phonemic glottal stop versus an epenthetic glottal stop; and the preceding word is lengthened or not (the presence/absence of final lengthening)
Description of data collection	Experiments involved pre-processing of the acoustic data for acquisition of acoustic data and acoustic analyses of glottal gestures and pre-processing of the raw output of the eye-tracking device (asc file) to timelines of fixations to interest areas for each trial.
Data source location	OSF Storage Frankfurt – Germany
Data accessibility	Repository name: Open Science Framework (osf.io)
	Data identification number: 10.17605/OSF.IO/PW74U
	Direct URL to data: 10.17605/OSF.IO/PW74U
	The data available in the Open Science Framework include the following:
	Experiment 1: Text files containing values from acoustic measurements, with html files explaining the variable names; Png files used as prompts for the production experiment.
	Experiments 2–4: Wave files used as stimuli for the experiments; text files with raw responses; html files (generated by R markdown) for the statistical analyses performed.
Related research article	Mitterer, H., Kim, S. & Cho, T. (2019). The glottal stop between segmental and suprasegmental processing: The case of Maltese. *Journal of Memory and Language*, 108, 104,034. DOI: https://doi.org/10.1016/j.jml.2019.104034

## Value of the data

• The data files contain trial-level data for all four experiments (an acoustic production experiment, a two-alternative forced choice task, a visual-world eye tracking task with printed target words and a gating task), allowing other researchers to apply other existing or forthcoming data-analysis techniques to these data.
• The data from the eye-tracking experiments contain the complete fixation history of each trial, allowing other researchers to use other statistical models such as growth-curve analysis [4] or general additive models [5] to test the time course of fixations or to test different time windows. Researchers may also examine the data from different perspectives to explore other aspects of the eye-tracking data as it fits with their research interests.
• By providing trial-level data, researchers can use additional covariates in the analysis that were of tangential interest to the primary research paper (such as usage data once there is a corpus of spontaneous Maltese).

## 1 Data description

The data files (raw UTF8 text) linked to this article contain trial level data for four experiments reported in [1]. We also provide meta-data in html files (generated from R markdown files) with the meaning of the different variable names, linking the variable names to the experimental factors. These html files also document the analysis reported in [1].

For Experiment 1, the data file contains information about the presence of the glottal gesture (as evident in the acoustic signal) and the type and duration of the glottal gesture when it is present (variables “firstSeg” and “dur”). (See [6] for more information about Maltese glottal stops.) It also contains information about the preceding context (“contextUsed”), the intended target word (“item”) and the duration of the other parts of the sentence (see the online meta-data files for details). Table 1 illustrates the first several lines of the text file containing the acoustic measurement data.

**Table 1:** Part of the text file that contains the information about acoustic measurements to illustrate the organization of the file. The variable names on the top row are explained in a meta-data html file in the OSF website (10.17605/OSF.IO/PW74U).

speaker	Item	Correct	contextInt	contextUsed	firstSeg	Dur	vowelDur	prevDur	nameDur	endDur	prevWordDur	hasPause	wordDur	LeOnset	sameVowel
S01	VInitial_01	1	il-kliem	il-kliem	V	0	70	100	666	730	404	0	340	6.43	−1
S01	VInitial_02	1	il-kelma	il-kelma	q	70	90	40	580	810	400	0	460	6.42	−1
S01	VInitial_03	1	il-kliem	il-kliem	V	0	100	90	710	680	400	0	399	6.36	−1
S01	VInitial_04	1	il-kelma	il-kelma	V	0	30	80	530	630	450	0	760	6.46	1
S01	VInitial_05	1	il-kliem	il-kliem	V	0	90	80	480	710	390	0	310	6.38	−1

For Experiment 2, the data file contains data for one trial on each line. Each line has information about the level of both the experimental factors (e.g., lengthening or no lengthening of the preceding word in the variable “case” and the amount of glottalization in the variable “step”) and the dependent variable of whether or not the participant heard a lexical (phonemic) glottal stop (in the variable “heardQ”). Additional variables indicate the block and the trial number as well as the reaction time (see meta-data provided with the data for additional details). Table 2 illustrates the first several lines of the raw response text file.

**Table 2:** Part of the text file that contains the raw response data for Experiment 2 to illustrate the organization of the file. The variable names on the top row are explained in a meta-data html file in the OSF website (10.17605/OSF.IO/PW74U).

case	wav	step	blockNumber	trialNumber	key	rt	Participant	heardQ
base	base_3.wav	3	0	0	left	11.74980987	pp01	0
acce	accent_2.wav	2	0	1	left	11.39967703	pp01	0
base	base_0.wav	0	0	2	left	2.283424109	pp01	0
acce	accent_1.wav	1	0	3	left	1.533297079	pp01	0

For Experiment 3, the data are distributed over three files. The first file contains trial-level data with the trial parameters and the behavioural reaction for all the trials (allTrials.txt): the click response with reaction time “rt” and exact location coded in two variables “*x*” and “*y*” for the x and y coordinate on the screen, and categorization data in relation to the four interest areas, “respCategory”. The second file contains the same information but only for trials with a correct response and a clear eye-tracking record (corrTrials.txt). This file is aligned by rows with the pre-processed eye-tracking data. The file “fixations.txt” contains the looks from 200 ms before the start of the critical words till 1400 ms after target onset in steps of 10 ms. The fixations have been pre-processed to indicate whether participants looked to one of the four objects (printed words) on the screen or on a neutral location (such as the middle of the screen). The online repository also contains a script for the further processing of these data into the time windows (that is, the data reduction of the eye-tracking data to one dependent variable) as reported in [1] and the script for the statistical analyses.

For Experiment 4, the data file contains trial level data on the independent variables (type of word, length of preceding word) and the dependent variable (whether the participants indicated to hear the word with a lexical (phonemic) glottal stop or not). Table 3 illustrates several lines of the text file with the raw response data. It also contains additional variables such as the exact item used on this trial as well as reaction time (see provided meta-data for details).

**Table 3:** Part of the text file that contains the raw response data for Experiment 4 to illustrate the organization of the file. The variable names on the top row are explained in a meta-data html file in the OSF website (10.17605/OSF.IO/PW74U).

pp	trialtype	Item	condition	wav	targetonse	targetw	competw	targetpos	competpos	Whereq	Trialno	RT	Key	heardQ
pp01	exp	qalb	q_Wd	David_Wd	566	qalb	Alpi	(1200,900)	(400,900)	2	10	801.87	Right	1
pp01	exp	qawwi	q_Wd	Jenny_Wd	585	qawwi	Awissu	(400,900)	(1200,900)	1	11	811.93	Left	1
pp01	exp	qanfud	q_Wd	David_Wd	566	qanfud	anzjan	(400,900)	(1200,900)	1	12	853.33	Left	1

## 2 Experimental design, materials, and methods

### 2.1 Participants

85 native speakers of Maltese participated in the experiments (16 (9 Female, 7 Male) in Experiment 1, 12 (9 Female, 3 Male) in Experiment 2, 41 in Experiment 3 (23 Female, 18 Male), and 16 (9 Female, 7 Male) in Experiment 4). All participants were in the age range between 18 and 28 years of age. The data reported here therefore reflect the linguistic behavior of the young Maltese speakers. They had normal hearing and normal or corrected-to-normal vision.

### 2.2 Apparatus

The experiments were performed in sound-attenuated booths at the Cognitive-Science lab of the University of Malta. Experiments were controlled by a standard PC using Speechrecorder^1^ for Experiment 1, PsychoPy (version 1.84) [3] for Experiment 2 and ExperimentBuilder (SR research) for Experiments 3 and 4. Vocal responses in Experiment 1 were recorded via a Focusrite CM25 large diaphragm condenser microphone connected to a Focusrite 2i2 USB audio interface that did the D/A conversion before storing the files on the computer. Keyboard and mouse presses were used in Experiments 2 through 4, while an SR Research Eyelink1000 eye tracker was used to additionally record eye movements in Experiment 3.

### 2.3 Materials and procedure

**Fig. 1:**
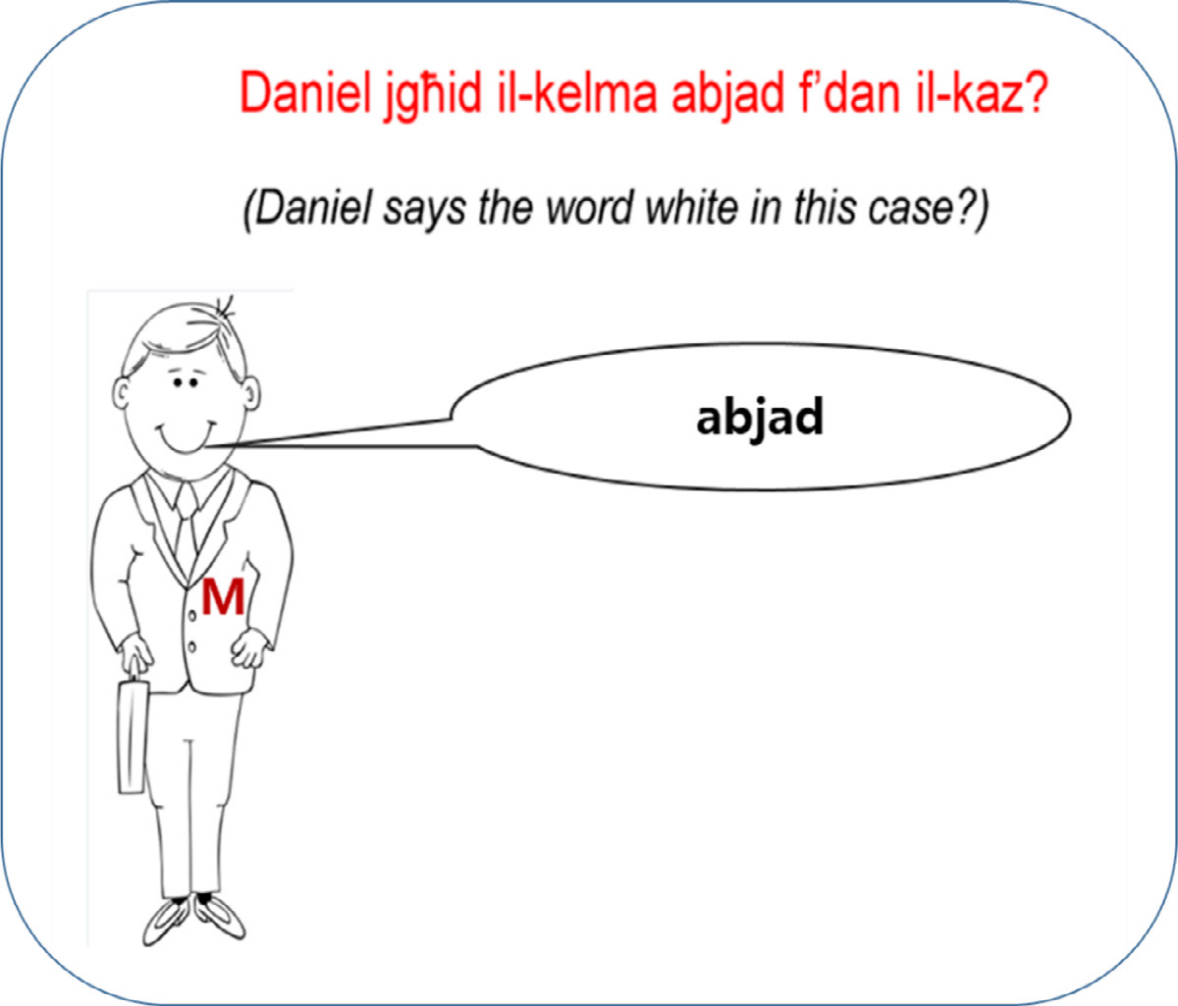
An example prompt re-drawn based on Fig. 1 used in Experiment 1 in [1] in which the actual cartoon character looked different from the one used here. The English translation is given here but was not shown in the actual experiment. Participants were instructed to answer the question based on the information provided in the picture. The speaker is the cartoon character “Matthew” (as marked by ‘M’), so that the correct answer is *Le, Matthew jgħid il-kelma abjad f'dan il-kaz* (Engl. ‘No, Matthew said the word ‘white’ in this case’). The critical word *abjad* /**ɑ**bjɑd/ (Engl. ‘white’) in this example is vowel-initial, and therefore it could potentially trigger a glottal-stop epenthesis.

For Experiment 1, the participants responded on 135 trials with data processed for the 70 experimental trials per participants; 35 trials with a unique vowel-initial test word and 35 trials with a unique glottal-stop initial test word (since no measures were taken on filler trials, those are not included in the data set). Critical words were elicited in a sentence-generation task with stimuli such as depicted in Fig. 1 (re-drawn based on Fig. 1 in [1]). The figure includes English translations not visible during the actual experiment. (See [1] for more detail on the elicitation procedure.)

The sentences recorded by the participants were then analysed using Praat [7] and a forced-alignment algorithm provided by the Munich Automatic Segmentation [8] online system. If the forced alignment found a glottal stop at the critical word juncture, the word was coded to have a glottal stop and the estimated duration of that glottal stop was used as a duration measure. If no glottal stop was found, a human coder (one of the authors) investigated whether there were cues for glottalization and if so, how long those cues were (a subset of the data was coded by a second-rater, which showed reasonable interrater reliability). Praat scripts were then used to read out the data from the textgrids to generate the presented data file.

For Experiment 2, the data were elicited by stimuli based on the sentence *tikteb il-kliem għam u nar* (Engl., ‘She writes the words he-swam and fire’; note that *he swam* is a single word in Maltese) recorded by an adult male speaker of Maltese. The parts preceding and following the critical word were spliced out to form a sentence frame. The preceding part was manipulated with PSOLA in Praat [6]. This algorithm allows to lengthen and shorten speech signals. It was used to generate two versions of the preceding part, one that had the same timing as the original, fluent utterance which was not produced with preboundary lengthening (i.e., lengthening of the preceding word) and one that was manipulated to be 55 ms longer than the original to emulate preboundary lengthening. This constitutes the first factor used to elicit the data with a test sentence with or without preboundary lengthening (cf., [9,10,11,12,13]).

For the second factor to elicit the data, the strength of the glottal gesture in the stimulus was generated with a target continuum over the initial 50 ms of the vowel-initial target word, originally produced without any phonetic evidence of glottalization. This was also done using PSOLA in Praat. Starting from the original utterance with no cues for glottalization, we added pitch and amplitude drops to mimic the typical properties of glottalized vowels [14]. Pitch was lowered from 100 to 60 Hz, and amplitude was lowered from 100% of the original to 50% of the original in 6 steps.

**Fig. 2:**
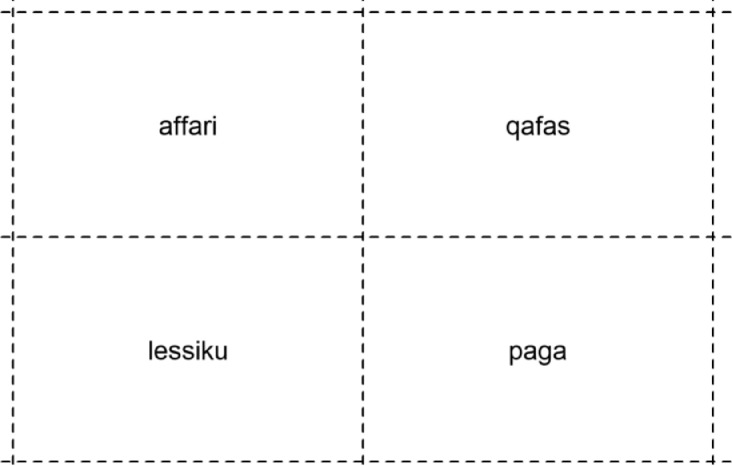
An example display that was used to generate the data for Experiment 3. The dotted lines are only for demonstration purposes and did not appear during the actual experiment.

For Experiment 3, participants were presented with a visual display of four words on a screen in the center of the screen's quadrants (see Fig. 2). At the same time, they heard a sentence in Maltese such as *Jenny tifhem qafas* (Engl., ‘Jenny understood frame’) with the instruction to click on the word that was “understood” (i.e., in this case the word *qafas*). All sentences had the structure of ‘Name understands word.’ Critically, on the experimental trials, the display that was presented to the participants contained both a glottal stop-initial word and a vowel-initial word. We labelled these two words as “pseudo-onset” overlap pairs [1]. In the example of Fig. 2, these are the words *qafas* /ʔɑfɑs/, Engl. ‘frame’, and *affari* /ɑf:ɑri/, Engl. ‘affair’. These words are phonetically similar in the onset except for the presence or absence of a phonemic glottal stop. Note that the two words are not typical onset-overlap pairs in which the first few speech sounds are the same (as in *beetle* versus *beaker*). That is, they differ in the first phoneme in their “dictionary form” (/ʔ/ in /ʔɑfɑs/ vs. /ɑ/ in /ɑf:ɑri/). However, the two words become overlap pairs if the vowel-initial word is produced with an epenthetic glottal stop (*affari* /ɑf:ɑri/ → [ʔɑf:ɑri]. If this glottal-stop epenthesis applies, the first three segments of *affarri* [ʔɑf:ɑri] are the same as in *qafas* /ʔɑfɑs/ → [ʔɑfɑs]. This is why we coined these pairs “pseudo-onset overlap pairs” to distinguish them from typical onset-overlap pairs. Typically, both members of such onset-overlap pairs attract the participant's visual attention in visual-world paradigms when the initial part of the target word is heard [15]. The experiment investigated to what extent this would be the case with these pseudo-onset overlap pairs, and whether it would be modified by a prosodic boundary (as reflected by the presence or absence of preboundary lengthening) before the critical target word.

There were two independent variables in Experiment 3: whether the phrase preceding the target word had preboundary lengthening or not (generated in the same way as in Experiment 2), and whether the target was the word with a (phonemic) glottal stop (which, in the example in Fig. 2, would be *qafas*) or the vowel initial word (which, in the example in Fig. 2, would be *affari*). There were 48 such pairs of pseudo-onset overlap pairs used in the experiment, a list of which can be found in Appendix B of [1].

In Experiment 4, the same materials as in Experiment 3 formed the basis for data collection. The stimuli were, however, shortened so that the participants heard no clear phonetic cues that might disambiguate the words in an overlap pair. For instance, in the case of the pseudo-onset overlap pair *affari-qafas*, participants heard [ʔɑf]), which is compatible with both the words *qafas* and *affari* when the latter was produced with an epenthetic glottal stop. Participants heard these “gated” [16] stimuli and were then asked to guess whether the speaker had intended the glottal stop-initial word (e.g., *qafas*) the vowel-initial word (e.g., *affari*) even when listeners did not hear the full word. That is, upon hearing a phonetic form of [ʔɑf], participants had to decide whether the speaker had intended *qafas* or *affari*. To make participants focus on the phonetic material and to prevent frustration on part of the participants, we used filler trials in which slightly more disambiguating cues were added. For example, in the instance of the pair *affari-qafas*, the participant heard the whole [f], which was phonologically longer in the word *affari* (where it is a geminate) than in the word *qafas* (where it is a singleton). Table A1 in [1] lists the cutting points for all 48 stimulus pairs. In the critical trials, participants had little phonetic information on the target word itself, so that the context (whether the preceding word was long or short) might make a difference.

## Declaration of Competing Interests

The authors declare that they have no known competing financial interests or personal relationships which have, or could be perceived to have, influenced the work reported in this article.

## Appendix Supplementary materials

Image, application 1
